# Beta-Lactams Therapeutic Monitoring in Septic Children–What Target Are We Aiming for? A Scoping Review

**DOI:** 10.3389/fped.2022.777854

**Published:** 2022-03-10

**Authors:** Ronaldo Morales Junior, Gabriela Otofuji Pereira, Gustavo Magno Baldin Tiguman, Vanessa D'Amaro Juodinis, João Paulo Telles, Daniela Carla de Souza, Silvia Regina Cavani Jorge Santos

**Affiliations:** ^1^Clinical Pharmacokinetics Center, School of Pharmaceutical Sciences, University of São Paulo, São Paulo, Brazil; ^2^Pediatric Intensive Care Unit, Department of Pediatrics, Hospital Sírio-Libanês, São Paulo, Brazil; ^3^Faculty of Pharmaceutical Sciences, State University of Campinas, São Paulo, Brazil; ^4^Department of Infectious Diseases, AC Camargo Cancer Center, São Paulo, Brazil; ^5^Pediatric Intensive Care Unit, University Hospital, University of São Paulo, São Paulo, Brazil

**Keywords:** therapeutic drug monitoring, beta-lactams, pharmacokinetics, pharmacodynamics, pediatrics, sepsis

## Abstract

The antimicrobial therapy of sepsis and septic shock should be individualized based on pharmacokinetic/pharmacodynamic (PK/PD) parameters to deliver effective and timely treatment of life-threatening infections. We conducted a literature scoping review to identify therapeutic targets of beta-lactam antibiotics in septic pediatric patients and the strategies that have been applied to overcome sepsis-related altered pharmacokinetics and increase target attainment against susceptible pathogens. A systematic search was conducted in the MEDLINE, EMBASE and Web of Science databases to select studies conducted since 2010 with therapeutic monitoring data of beta-lactams in septic children. Last searches were performed on 02 September 2021. Two independent authors selected the studies and extracted the data. A narrative and qualitative approach was used to summarize the findings. Out of the 118 identified articles, 21 met the eligibility criteria. Population pharmacokinetic modeling was performed in 12 studies, while nine studies reported data from bedside monitoring of beta-lactams. Most studies were conducted in the United States of America (*n* = 9) and France (*n* = 5) and reported PK/PD data of amoxicillin, ampicillin, azlocillin, aztreonam, cefazolin, cefepime, cefotaxime, ceftaroline, ceftazidime, doripenem, meropenem and piperacillin/tazobactam. Therapeutic targets ranged from to 40% *f*T> MIC to 100% *f*T> 6 × MIC. Prolonging the infusion time and frequency were most described strategies to increase target attainment. Monitoring beta-lactam serum concentrations in clinical practice may potentially maximize therapeutic target attainment. Further studies are required to define the therapeutic target associated with the best clinical outcomes.

## Introduction

Approximately 30% of the children admitted to the pediatric intensive care unit (PICU) are affected by pediatric sepsis and septic shock, which are associated with high mortality and morbidity rates (1, 2). These diagnoses require early and appropriate antibiotic therapy in order to achieve the best outcome (3).

Beta-lactams are the most frequently prescribed antibiotics for critically ill septic patients. From a pharmacokinetic and pharmacodynamic (PK/PD) perspective, beta-lactams exhibit time-dependent antibiotic activity, that is, the time that free serum concentrations remain above the minimum inhibitory concentration (MIC) as a function of the dosing interval (% *f*T> MIC) is a surrogate marker of effectiveness (4). Nonetheless, an international multicenter survey demonstrated that there is an important heterogeneity of targets routinely used in patients > 18 years admitted into ICUs (5).

Pediatric patients often represent a challenge to obtain adequate serum concentrations of antibiotics due to the high variability in body composition, renal and hepatic clearance, or even in the maturation of biotransformation enzymes, resulting in distinct patterns of drug absorption, distribution, and elimination (6, 7). In addition, hydrophilic antibiotic as beta-lactams are largely influenced by the systemic inflammatory response syndrome (SIRS), use of vasopressors, fluid therapy, sepsis-induced organ dysfunctions and by other pathophysiologic factors of critical illness (8, 9).

Availability of information on bacterial susceptibility is also problematic. When the MIC of the isolated strain is not available, it is common to consider the epidemiological cut-off (ECOFF) or the susceptibility breakpoint of the presumed/identified pathogen (10). However, it is possible that patients have infections caused by pathogens with lower MICs than these thresholds.

While the PK/PD target is not consensually defined for beta-lactams, we conducted a literature scoping review to identify which therapeutic targets have been used in the last 10 years in septic pediatric patients. The secondary objectives were to describe the strategies that have been applied to increase the percentage of target attainment (PTA) of empiric therapy against susceptible pathogens.

## Methods

### Protocol and Registration

The protocol for this scoping review was registered in the Open Science Framework (OSF) platform: doi: 10.17605/OSF.IO/9K3D8.

### Eligibility Criteria

Primary studies of septic pediatric patients, from birth to 18 years of age, that involve beta-lactam therapy with measured serum concentrations (including PK and PK/PD data) were eligible for inclusion. Sepsis is described as presumed or proven infection with life-threatening organ dysfunction (11). However, since the most adequate criteria to define organ dysfunction in children have not yet been defined (3), we chose not to require a specific definition for the purpose of this review, including all studies describing the population as septic. We excluded studies that involved animals, drug classes other than beta-lactams and populations other than pediatric. In the case of studies with a mixed population (adult and pediatric), we considered only the data referring to pediatric patients. Conference abstracts, review articles and letters to the editor were considered ineligible, but their references were evaluated for additional relevant studies.

### Information Sources and Searches

The searches were conducted in the MEDLINE (via PubMed), EMBASE and Web of Science databases (Table 1). Searches were limited to studies available in English, Portuguese and Spanish published since 2010 to map the most updated evidence. Whenever possible, MeSH and Emtree terms and keywords were used to increase the chances of capturing more studies. Additionally, reference lists from retrieved articles and reviews were examined for additional relevant studies not retrieved from our search. The search strategies for each database are presented in Table 1. Last searches were performed on 02 September 2021.

**Table 1:** Search strategy.

**MEDLINE *via* Pubmed**
(((pediatric[MeSH Terms]) OR (child[MeSH Terms]) OR (children[MeSH Terms]) OR pediatri* OR paediatr*) AND ((sepsis[MeSH Terms]) OR (septicemia[MeSH Terms]) OR (septic shock[MeSH Terms])) AND ((beta lactam[MeSH Terms]) OR (beta-lactam[MeSH Terms]) OR (β-lactam[MeSH Terms])) AND ((therapeutic drug monitoring[MeSH Terms]) OR (drug monitoring[MeSH Terms]) OR (drug concentration) OR (plasma concentration) OR (serum concentration))).
**EMBASE**
('child'/exp OR pediatri* OR paediatr*) AND ('sepsis'/exp OR 'septicemia'/exp OR 'septic shock'/exp) AND 'beta lactam'/exp AND ('drug monitoring'/exp OR 'drug concentration' OR 'plasma concentration' OR 'serum concentration').
**Web of science**
(((pediatric) OR (child) OR (children) OR pediatri* OR paediatr*) AND ((sepsis) OR (septicemia) OR (septic shock)) AND ((beta lactam) OR (beta-lactam) OR (β-lactam)) AND ((therapeutic drug monitoring) OR (drug monitoring) OR (drug concentration) OR (plasma concentration) OR (serum concentration))).

### Selection of Sources of Evidence

Two reviewers independently screened titles and abstracts for inclusion; those potentially eligible were then reviewed in full text. Discrepancies between the reviewers were resolved by consensus. Study selection was performed using the Rayyan software (https://www.rayyan.ai/).

### Data Charting Process and Items

Two researchers independently charted data from the selected studies using a standardized Microsoft Excel spreadsheet; any discrepancies were resolved by consensus. The following data were collected: main author, year, country/countries from which study participants were recruited, study design, subject characteristics, number of subjects, renal function, antibiotics used, therapeutic target considered for population pharmacokinetics analysis or therapeutic drug monitoring (TDM), the susceptibility MIC breakpoint considered, dosing recommendations (when applicable) and main findings/conclusions. A narrative and qualitative approach was used to summarize the findings due to the high heterogeneity between the included studies.

## Results

**Figure 1:**
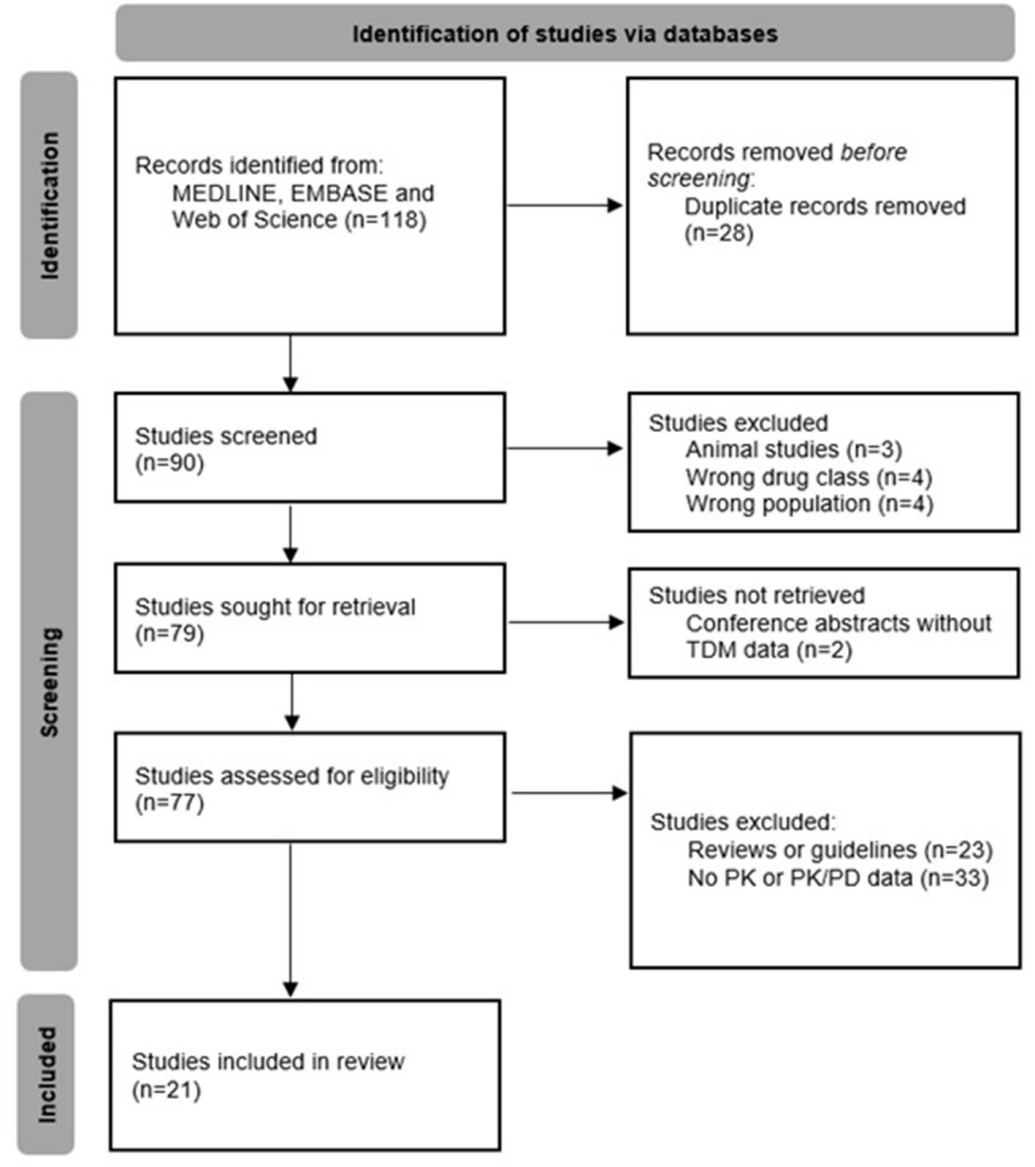
Flowchart describing the study selection process.

Our literature search yielded 118 records, of which 28 were removed as they were duplicates. The remaining 90 articles were screened for eligibility; in total, 21 studies were included in this review (Figure 1).

Studies were published between 2014 and 2021 and all of them were available in English language; most (57%) were population-based PK studies. The number of subjects varied from 1 (case reports) to 1.272, and 13 different antibiotics were investigated. Table 2 summarizes their characteristics and main findings.

**Table 2:** Characteristics of the included papers.

**Citation**	**Region**	**Study design**	**Subjects characteristics**	**No. subjects**	**Median ClCr (ml/min/1.73 m^**2**^) or Cr (mg/dL)**	**Antibiotic**	**Therapeutic target**	MIC^*^	**Dosing recommendations**	**Strategy applied or suggested to increase the PTA**
De Cock et al. (12)	Belgium	Population PK study	ICU Patients between 1 month and 15 years	50	0.21 mg/dL	Amoxicillin-clavulanic acid	40%*f*T>MIC	8 mg/L	25 mg/kg q4h (1 h-infusion)	To shorten the interval and extend infusion duration
Mir et al. (13)	Pakistan	Prospective cohort	Infants <59 days of age	20	NR	Amoxicillin	50%*f*T>MIC	2 mg/L	75–100 mg/ kg/day (oral)	To increase the dose
Ericson et al. (14)	United States of America	Retrospective cohort	Infants <28 days of age	1272	0.2–2.5 mg/dL	Ampicillin	50%*f*T>MIC	8 mg/L	≥75mg/kg/dose every 6 or 8 h)	To increase the dose and shorten the interval
Wu et al. (15)	China	Population PK study	preterm and term infants ≤ 72h old	45	NR	Azlocillin	70%*f*T>MIC	8 mg/L	100 mg/kg q6-8h (0.5h-infusion)	To shorten the interval
Cies et al. (16)	United States of America	Population PK study	ICU patients between 9 months and 6 years	13	NR	Piperacillin-tazobactam	50%*f*T>MIC	16 mg/L	100 mg/kg q6h (3h- infusion); 400 mg/kg/day CI	To extend infusion duration
Nichols et al. (17)	United States of America	Population PK study	ICU patients between 9 months and 11 years	12	103 mL/min/1.73 m^2^	Piperacillin-tazobactam	(a) 50%*f*T>MIC (b) 100%*f*T>MIC	16 mg/L	(a) 80-100 mg/kg q8h (4h-infusion) (b) None of the tested regimens	To extend infusion duration
De Cock et al. (18)	Belgium	Population PK study	ICU patients between 1 month and 15 years	47	109 mL/min/1.73 m^2^	Piperacillin-tazobactam	60%*f*T>MIC	16 mg/L	75-100 mg/kg q4h (1-2h-infusion); 300 mg/kg/day CI	To increase the dose and shorten the interval
Beranger et al. (19)	France	Population PK study	All children aged <18 years	50	142 mL/min/1.73 m^2^	Piperacillin-tazobactam	(a) 50%*f*T>MIC (b) 50%*f*T>4x MIC (c) 100%*f*T>MIC (d) 100%*f*T>4x MIC	16 mg/L	(a) 75-100 mg/kg q6–8 (3–4 h-infusion) (b) NR (c) 75–100 mg/kg/day CI (d) NR	To extend infusion duration
Chongcharoenyanon et al. (20)	Thailand	Prospective randomized trial	ICU Patients between 1 month and 18 years	90	NR	Piperacillin-tazobactam	(a) 50%*f*T>4 × MIC (b) 50%*f*T>MIC	16 mg/L	100 mg/kg q8 h (4 h-infusion)	To extend infusion duration
Leroux et al. (21)	France	Population PK study	Neonates and young infants (postmenstrual age under 44 weeks)	100	0.49 mg/dL	Cefotaxime	75%*f*T>MIC	2 mg/L	50 mg/kg q6–12 h (0.25–0.5 h infusion)	To shorten the interval
Beranger et al. (22)	France	Population PK study	All children aged <18 years	49	171 mL/min/1.73 m^2^	Cefotaxime	(a) 100%*f*T>4 × MIC (b) 100%*f*T>MIC	0.5 mg/L	100 mg/kg/day CI	To extend infusion duration
Hartman et al. (23)	Netherlands	Secondary analysis of a randomized controlled trial	ICU Patients between 1 month and 18 years	37	0.46 mg/dL	Cefotaxime	(a) 100%*f*T>4 × MIC (b) 100%*f*T>MIC	2 mg/L	NR	None
Bui et al. (24)	France	Population PK study	ICU Patients between 28 days and 12 years	108	198 mL/min/1.73 m^2^	Ceftazidime	(a) 60%*f*T>MIC (b) 90%*f*T>MIC	8 mg/L	90-150 mg/kg/day CI	To extend infusion duration
Li et al. (25)	China	population PK study	Neonates and young infants (postmenstrual age under 48 weeks)	146	0.38 mg/dL	Ceftazidime	70%*f*T>MIC	8 mg/L	25–30 mg/kg q8/12 (infusion duration NR)	To increase the dose and shorten the interval
Yasmin et al. (26)	United States of America	Case report	4-year-old child	1	77.4 mL/min/1.73 m^2^	Ceftazidime-Avibactam and aztreonam	100%*f*T> MIC	8 mg/L	NR	TDM and antibiotic synergy
Cies et al. (27)	United States of America	Case series	ICU Patients between 1 year and 13 years	7	>60 mL/min/1.73 m^2^	Ceftaroline	40%*f*T>4–6 × MIC	2 mg/L	NR	TDM
Cies et al. (28)	United States of America	Case report	2-year-old girl	1	157 mL/min/1.73 m^2^	Meropenem	40%*f*T>MIC	8 mg/L	NR	TDM
Cies et al. (29)	United States of America	Population PK study	ICU Patients between 1 year and 15 years	9	168 mL/min/1.73 m^2^	Meropenem	(a) 40%*f*T>MIC (b) 80%*f*T>MIC	2 mg/L	40 mg/kg q6–8 h (3-4h infusion); 120 mg/kg q24 h CI	To extend infusion duration
Cies et al. (30)	United States of America	Case report	16-year-old boy	1	NR	Aztreonam	40%*f*T>MIC	16 mg/L	NR	To extend infusion duration
Cies et al. (31)	United States of America	Retrospective cohort	All children aged <18 years	82	>60 mL/min/1.73 m^2^	Ampicillin, cefazolin, cefepime, cefotaxime, ceftaroline, doripenem, meropenem, piperacillin/tazobactam	(a) 40%*f*T>4–6 × MIC (b) 40%*f*T>MIC (c) 100%*f*T>MIC	NR	NR	TDM

* *Susceptibility breakpoint considered by the study*.

*PK, pharmacokinetics; PD, pharmacodynamics; ICU, intensive care unit; PTA, percentage of target attainment; TDM, therapeutic drug monitoring; fT>MIC, time that free concentrations remain above the minimum inhibitory concentration (MIC) as a function of the dosing interval; NR, not reported; CI, continuous infusion*.

The most described strategies that were applied to increase the PTA were: extending infusion duration (*n* = 10), shortening dose interval (*n* = 6), TDM (*n* = 4) and increasing dose (*n* = 3).

### Penicillins

De Cock et al. investigated amoxicillin-clavulanic acid PTA with target efficacy defined as 40% *f*T> MIC against strains with MIC of 8 mg/L for amoxicillin. With commonly prescribed doses (25 mg/kg every 6–12 h), target attainment was estimated at 48–73%. The authors demonstrated that more frequent doses (25 mg/kg every 4 h) achieve higher PTA: 96–99%. They also reported that a 1-h infusion was superior to bolus dosing for patients with augmented renal clearance (12).

Mir et al. considered a target of 50% *f*T> MIC to investigate target attainment in patients receiving high doses of oral amoxicillin (75–100 mg/ kg/day). The resultant concentrations exceeded the susceptibility breakpoint for *S*. pneumoniae strains (2 mg/L) (13). Ericson *et al*. conducted a large cohort with 1,272 infants receiving ampicillin and they also considered a target of 50% *f*T> MIC. *Streptococcus agalactiae* (MIC 0.25 mg/L) was the most frequent isolated pathogen (>60%). They found that achieving this target was associated with decreased duration of bacteremia, which occurred more frequently with patients who received high ampicillin doses with a short dosing interval (≥75 mg/kg/dose every 6 or 8 h) (14).

In a recent phase II clinical trial of azlocillin in preterm and term infants ≤ 72 h old, Wu et al. conducted Monte Carlo simulations with a desired target of 70% *f*T> MIC up to 32 mg/L. The authors showed that increasing the frequency (100 mg/kg every 8 h) achieved the target in 91.2% of infants compared to 63.1% of PTA with 100 mg/kg, every 12 h, both with a 0.5 h infusion (15).

Five studies reported PK/PD data of piperacillin (16–20). Chongcharoenyanon et al. designed a randomized controlled trial to compare piperacillin target attainment between extended infusion and intermittent bolus methods in children. Those who received extended infusion had a higher proportion of patients who achieved 50% *f*T>MIC [90.9% vs. 60.0% (*P* = 0.11)] and 50% *f*T>4x MIC [72.7% *vs*. 30.0% (*P* = 0.06)], but the authors could not demonstrate significant differences in clinical outcomes between groups (20). The other four studies used a population pharmacokinetics approach to evaluate PTA of different simulated dosing regimens (16–19). They considered a minimum target of 50% to 60% *f*T> MIC and suggested extend infusion duration (2–4 h) to maintain plasma concentrations above the susceptibility breakpoint of *Pseudomonas aeruginosa* (16 mg/L). Nichols et al. also carried out Monte Carlo simulations considering 100% *f*T> MIC, while Beranger et al. tested four different targets: 50% *f*T> MIC, 50% *f*T> 4x MIC, 100% *f*T> MIC and 100% *f*T> 4x MIC. For these higher targets, not even extended infusion regimens were sufficient, but only when piperacillin was administered by continuous infusion (17, 19).

### Cephalosporins

Leroux et al. considered a target of 75% fT> MIC for cefotaxime analysis in neonates and infants. Empiric regimen underdosed septic older newborns with only 53–68% of PTA against pathogens with MICs > 2 mg/L, so the authors have established 50 mg/kg twice a day to four times a day according to gestational age and postnatal age (21). Béranger et al. and Hartman et al. defined higher targets of 100% *f*T> MIC and 100% *f*T> 4x MIC. They only found satisfactory target achievement against strains with low MIC ( ≤ 0.5 mg/L) with usual dose regimens of 100–150 mg/kg/day and suggested applying continuous infusion in septic children (22, 23).

We identified two population pharmacokinetics studies with ceftazidime (24, 25). Li et al. set the target to 70% *f*T> MIC to test PTA of initial doses in neonates against pathogens with MIC up to 8 mg/L. Simulations with the initial dose of 25 mg/kg twice daily achieve the target only for newborn with postnatal age ≤ 3 days. They proposed higher initial doses of 30–40 mg/kg every 8 h for optimal PTA (25). Bui et al. studied older patients (up to 12 years) and considered the targets of 60 and 90% *f*T> MIC. Simulations with ceftazidime recommended dose of 150 mg/kg/day as 0.5 h-intermittent infusion resulted in PTA of 48.3% in patients with cystic fibrosis, compared to 75.4% in other children (for 60% *f*T> MIC). For the same MIC and dosing, continuous infusion enabled more than 99% of all patients to reach the target (24).

Ceftazidime-avibactam was cited in one case report. A 4-year-old immunocompromised child, who developed a bloodstream infection by *Enterobacter hormaechei* producing *Klebsiella pneumoniae* Carbapenemase−4 and New Delhi metallo-β-lactamase, was successfully treated with antibiotic synergy of ceftazidime-avibactam and aztreonam. The authors measured serum concentrations and considered a target of 100% *f*T> MIC for both agents to perform TDM analysis (26).

Cies et al. published TDM outcomes in 7 septic patients receiving ceftaroline 15 mg/kg every 6 h. They considered a target of 40% fT> 4–6x MIC for doses adjustments against *Staphylococcus spp*. (MIC ≤ 2 mg/L). The patients presented different PK changes compared to healthy data and all of them had adjustments made to their dosing regimens to meet the PK/PD endpoint against isolated methicillin-resistant *Staphylococcus aureus* (MRSA). All patients achieved a positive microbiological and clinical response (27).

### Carbapenems and Monobactam

A TDM case report of meropenem described the clinical course of a 2-year-old girl with *Serratia marcescens* (meropenem MIC < 0.25 mg/L) ventriculitis. The target was set to 40% *f*T> MIC and the initial dose of 40 mg/kg every 6 h with 0.5 h-intermittent infusion was insufficient. The meropenem regimen was changed to a continuous infusion of 200 mg/kg/day and then the serum trough level and cerebrospinal fluid levels were enough for cerebrospinal fluid sterilization and successful clinical outcome (28).

Cies et al. considered the target of 40% *f*T> MIC and also 80% *f*T> MIC to investigated PTA of different infusion durations with 120–160 mg/kg/day of meropenem. For the first one, the 3 h-extended infusion provide appropriate exposures against susceptible pathogens. When considering 80% *f*T> MIC, continuous infusion was needed (29).

A TDM analysis of aztreonam was reported in a case of a 16-year-old tetraplegic boy with history of cervical spinal cord injury, chronic respiratory failure, and tracheostomy, hospitalized for empyema caused by Pseudomonas aeruginosa only sensitive to aztreonam (MIC of 2–6 mg/L). The authors established a target of 40% fT> MIC and the initial dose regimen of 2 g every 6 h of aztreonam required prolonged infusions over 4 h to reach the target. The patient presented further negative cultures (30).

### Mixed

A bedside monitoring cohort study published by Cies et al. aimed to verify whether commonly prescribed beta-lactam dosing regimens achieve 40% *f*T > 4–6× MIC in critically ill children, which is the most used target at their institution. They observed that 73 of 82 patients (95%) had subtherapeutic concentrations and dose adjustments (e.g., dosing interval shortening or prolonging infusion duration) were made within 48 h of beta-lactam initiation. The authors also have established an upper limit of 100% *f*T > 6× MIC and 5 patients (6.4%) presented supratherapeutic concentrations and had reductions in their dosing regimens. After individual dose adjustments, all patients with a positive culture achieved microbiological response with eradication of isolated pathogens (31).

## Discussion

Our scoping review of the literature has identified diverse therapeutic targets for beta-lactams in PK studies involving septic pediatric patients, ranging from to 40% *f*T> MIC to 100% *f*T> 6× MIC. The empiric recommended doses of beta-lactams commonly fail to achieve the target against less susceptible pathogens, and the most described strategies to increase target attainment were increasing dosing or frequency of administration and extending the infusion time. Furthermore, monitoring beta-lactam serum concentrations in clinical practice is described as a valuable tool to overcome PK variability of critically ill patients through individual dosing adjustments.

None of the included studies were designed to identify whether reaching higher targets leads to reduced morbidity or mortality. The study conducted by Ericson et al. found that ampicillin concentrations above the MIC for ≥50% of the dosing interval was associated with decreased duration of bacteremia, but no broad spectrum antibiotics were investigated (14). Data from prospective and randomized clinical trials with cefepime and ceftazidime in septic adults showed significant greater clinical cure rates and bacteriological eradication when patients achieved 100% *f*T> MIC compared with those who did not (32). The DALI study, a multinational point-prevalence study with beta-lactams, also found that adult patients achieving 100% *f*T> MIC are more likely to have positive clinical outcomes with lower levels of sickness severity (33). Recent guidelines suggest that 100% *f*T> MIC is a reasonable target for critically ill patients, mostly based on adult studies, although even higher targets of 100% *f*T> 4–10x MIC have been used in clinical practice (10, 34). On the other hand, PK/PD parameters of newer antibiotics (e.g., ceftolozane-tazobactam) have been investigated phase-3 studies and converged with targets <100% *f*T> MIC (35, 36).

Although most studies have suggested that beta-lactams are usually under-dosed, supratherapeutic beta-lactam concentrations may also have negative consequences. Only one study, conducted by Cies et al., defined the threshold for beta-lactam concentrations to reduce the risk of toxicity, with an upper limit of 100% *f*T > 6× MIC (31), while a previous adult trial trough considered trough levels up to 10× MIC (37). Neurotoxicity has the strongest association with elevated beta-lactams concentrations (38–41), but no study of this review described any neurotoxicity case. Other previously described beta-lactams-associated adverse reactions as nephrotoxicity, hepatotoxicity and cytopenias seem to be concentration independent and they have low incidence in pediatric patients (42).

The Surviving Sepsis Campaign guidelines for the management of septic shock and sepsis-associated organ dysfunction in children recommends individualizing antimicrobial therapy based on PK/PD principles to provide more effective and safer treatments of life-threatening infections (3). This includes considering the potential PK changes resulting from inflammatory response and/or organ dysfunction for initial dosing selection, applying different administration techniques such as once-daily dosing or prolonged infusion and dosing adjustments based on serum concentrations and individual PK parameters estimated with kinetic equations or Bayesian softwares (43). Most authors of the included articles recommended extending the infusion time (which includes prolonged and continuous infusions) and increasing dosing frequency to increase the PTA of beta-lactams (12, 16, 17, 19, 24, 29, 44). Chongcharoenyanon et al. demonstrated in a prospective, randomized trial that septic children receiving extended infusion of piperacillin present significantly higher target attainment and they recommended this approach should be implemented in clinical practice (20).

The bedside monitoring cohort study of Cies et al. impressively found that 95% of the children receiving beta-lactams were outside of the therapeutic window and it reinforces the difficulty of reaching the therapeutic target with general dosing recommendations for patients who have wide inter and intra-individual pharmacokinetic variation (31). Although beta-lactams TDM is not widely applied (5), the cases reported by Cies and Yasmin et al. demonstrate the usefulness of verifying beta-lactams concentration in clinical practice to optimize the doses in clinical practice (26–28, 30). Unfortunately, commercially assays are not yet available for beta-lactams as there are for aminoglycosides and glycopeptides and few hospitals can perform high-performance liquid chromatography or mass spectrometry essays (34). Others barriers to widespread incorporation of beta-lactam TDM into clinical practice are insufficient knowledge among health care providers, lack of cost-effectiveness evaluation and, as cited above, availability of bacterial susceptibility information (45).

Guidelines to standardize beta-lactam TDM practices are necessary but further randomized clinical trials are still required to define which therapeutic targets are associated with the best bacteriological and clinical responses in pediatric patients. The toxic thresholds for beta-lactams also require additional investigations.

Our scoping review presents some limitations. Although we implemented a systematic approach to map the current and updated evidence on beta-lactams TDM in pediatric septic patients, some studies might have been missed if they were not indexed in the searched databases, published in languages other than English, Spanish and Portuguese or before 2010. As we have not standardized the criterion for the diagnosis of sepsis, our review may have included studies with different definitions of sepsis and it limits the compilation of our findings. However, the possible variation in the sepsis definitions of each study probably did not influence the choice of the therapeutic target used. Publication bias was possible as some studies may not have been published due to negative results. This review has not assessed the methodological quality of the studies as scoping reviews aim to identify, map and describe all the available evidence regardless of their quality.

## Conclusion

Our scoping review has identified a wide range of therapeutic targets for beta-lactams in pediatric studies, from to 40% *f*T> MIC to 100% *f*T> 6× MIC. None of the included studies have identified effects of target attainment on morbidity or mortality and further randomized controlled trials are required to define the therapeutic target associated with the best clinical outcomes. The main described strategies to increase the PTA of empirical doses against sensitive pathogens were to increase the infusion duration or dosing frequency. Due to the unpredictability of pharmacokinetics in children and the pathophysiological changes during sepsis, the implementation of beta-lactam TDM may potentially be a valuable tool to maximize therapeutic target attainment.

## Author Contributions

RM, GP, and GT contributed to the study design, performed the literature review, screened titles and abstracts, and extracted data from the selected studies. VJ and JT collaborated in writing and editing the manuscript. DS and SS reviewed the manuscript. All authors have read and agreed to the published version of the manuscript.

## Conflict of Interest

The authors declare that the research was conducted in the absence of any commercial or financial relationships that could be construed as a potential conflict of interest.

## Publisher's Note

All claims expressed in this article are solely those of the authors and do not necessarily represent those of their affiliated organizations, or those of the publisher, the editors and the reviewers. Any product that may be evaluated in this article, or claim that may be made by its manufacturer, is not guaranteed or endorsed by the publisher.
